# Papillary Thyroid Carcinoma Arising Within a Mature Ovarian Cystic Teratoma: A Case Report

**DOI:** 10.1155/crie/7914933

**Published:** 2025-07-24

**Authors:** Pakaworn Vorasart, Rangsima Aroonroch, Naparat Rermluk, Orawin Vallibhakara, Chutintorn Sriphrapradang

**Affiliations:** ^1^Somdech Phra Debaratana Medical Center, Faculty of Medicine Ramathibodi Hospital, Mahidol University, Bangkok 10400, Thailand; ^2^Department of Pathology, Faculty of Medicine Ramathibodi Hospital, Mahidol University, Bangkok 10400, Thailand; ^3^Department of Obstetrics and Gynecology, Faculty of Medicine Ramathibodi Hospital, Mahidol University, Bangkok 10400, Thailand; ^4^Department of Medicine, Faculty of Medicine Ramathibodi Hospital, Mahidol University, Bangkok 10400, Thailand

**Keywords:** decision-making, dermoid cyst, neoplastic cell transformation, ovarian neoplasms, teratoma, thyroid neoplasms

## Abstract

**Introduction:** Mature cystic teratoma is a common benign ovarian germ cell tumor containing well-differentiated cells from three germ layers. Malignant transformation within these teratomas, such as papillary thyroid carcinoma, is extremely rare.

**Case Report:** A 62-year-old asymptomatic woman was found to have a 5 cm hyperechoic lesion with an internal cystic component in her left ovary, suspected to be a mature teratoma. A total hysterectomy with bilateral salpingo-oophorectomy was performed, removing an unruptured, thin-walled ovarian tumor. Gross pathology revealed a uni-loculated solid-cystic lesion with smooth serosa, a homogenous tan solid part containing soft tan hair, and no papillary projections, adhesions, or ascites. Pathology identified a 2 cm papillary thyroid carcinoma (classic subtype) arising in a 4.7 cm mature teratoma, without lymphovascular invasion or ovarian surface involvement. Thyroid ultrasound, thyroid function tests, and PET imaging showed no abnormalities or metastasis. The role for total thyroidectomy and radioactive iodine ablation was discussed. After reviewing the pathology and confirming the absence of aggressive tumor behavior, shared decision-making led to opting against further treatment. Three years postoperatively, there was no recurrence or metastasis.

**Conclusions:** This case describes the rare occurrence of papillary thyroid carcinoma within a mature ovarian teratoma. Currently, there is a lack of consensus on postoperative management. In selected cases with no evidence of metastasis or aggressive features, conservative management may be a reasonable option after thorough evaluation.

## 1. Introduction

Mature cystic teratomas, commonly known as dermoid cysts, are the most common germ cell tumors of the ovary, accounting for ~20% of ovarian tumors [1]. These tumors are typically benign, and contain mature tissue elements derived from all three germ layers: ectodermal (skin, hair follicles, and sebaceous glands), mesodermal (muscle, bone, cartilage, or fat), and endodermal (mucinous or ciliated epithelium) origins [2, 3]. They most commonly affect women between the ages of 30 and 40 [4] but may occur at any age. However, malignant transformation of tissue components within mature ovarian teratomas is extremely rare, with reported rates ranging from 0.17% to 2% [5–7], and most commonly observed in postmenopausal women [7].

When malignant transformation occurs, treatment must be tailored to the specific transformed histology [6]. Squamous cell carcinoma is the most common histologic variant of malignant transformation, accounting for about 80% of cases [8]. Less common malignancies include thyroid carcinomas, adenocarcinomas, sarcomas, malignant melanomas, basal cell carcinomas, sebaceous tumor, and carcinoid tumors. Even rarer are cases of a single mature cystic teratoma containing multiple types of malignant transformations.

We present a rare case of papillary thyroid carcinoma identified within a mature cystic teratoma of the ovary. This case highlights the diagnostic challenge in differentiating metastatic thyroid carcinoma to the ovary from malignant transformation of a teratoma into papillary thyroid carcinoma. Given the limited evidence on optimal management strategies for this rare condition, we provide a comprehensive review of the current literature, and discuss evidence-based approaches for managing such cases.

## 2. Case Presentation

**Figure 1:**
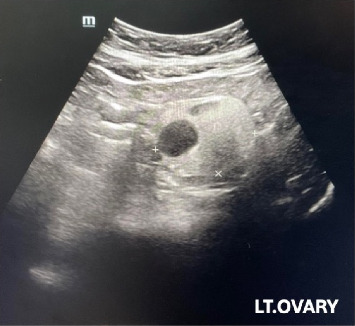
Ultrasound of the abdomen showing a 5 cm hyperechoic lesion with an internal cystic component in the left ovary.

During a check-up, a 62-year-old postmenopausal asymptomatic woman was found to have a 5 cm hyperechoic lesion with an internal cystic component in her left ovary (Figure 1), suspected to be a mature cystic teratoma. A transvaginal ultrasound confirmed the presence of a thin-walled cystic lesion at the left ovary, without any solid components. Preoperative serum levels of CA125, HE4, CA19-9, and CEA were all within normal limits. A preliminary diagnosis of mature cystic teratoma was made. The patient subsequently underwent total hysterectomy with bilateral salpingo-oophorectomy, during which an unruptured thin-walled ovarian tumor was removed.

**Figure 2:**
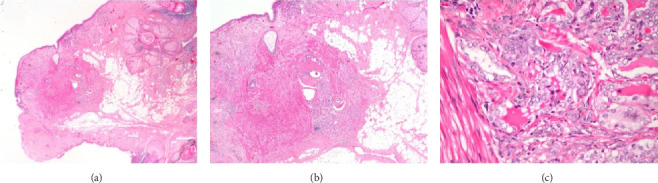
Pathology. (A) Focus of papillary thyroid carcinoma arising within a mature cystic teratoma (H&E, 20x), (B) higher magnification of papillary thyroid carcinoma within a mature cystic teratoma (H&E, 40x), and (C) papillary thyroid carcinomatous part is characterized by clusters of thyroid follicles, lined by cells with overlapping, clear nuclei, and irregular nuclear membranes (H&E, 400x).

Gross pathological examination revealed a uniloculated solid-cystic lesion with a smooth serosal surface. The solid portion showed a homogenous, soft, and tan appearance with hair. There were no ascites, papillary projections, or adhesions observed between the tumor, omentum, and uterus. Histopathological report identified a 2 cm classic subtype of papillary thyroid carcinoma arising within a 4.7 cm mature teratoma, without lymphovascular invasion or involvement of the ovarian surface (Figure 2). Thyroid ultrasound and thyroid function tests were normal (thyroid-stimulating hormone [TSH] 1.29 mIU/L [reference range, 0.35–4.94] and free T4 0.88 ng/dL [reference range, 0.7–1.48]), and PET imaging showed no evidence of metastasis. Serum thyroglobulin was not measured, as the presence of an intact native thyroid gland would limit its interpretive value in this setting, and would not have influenced the management plan.

The role for a total thyroidectomy and radioactive iodine ablation was discussed. However, after reviewing the pathology and confirming the absence of aggressive behavior in the papillary thyroid carcinoma, a shared decision was made not to pursue further management. During a 3-year follow-up, no recurrence or metastasis of the tumor was detected on computed tomography.

## 3. Discussion

Thyroid tissue, originating from endodermal cells, is found in 5%–20% of mature cystic teratomas [9, 10]. When thyroid tissue constitutes more than 50% of the teratoma, the lesion is defined as struma ovarii. Malignant transformation of thyroid tissue within a mature cystic teratoma is rare, with an estimated incidence of 0.1%–0.3% of cases [11]. Among these, papillary thyroid carcinoma is the most commonly reported histologic type [12–14], frequently occuring in its classical and follicular subtypes, similar to those found in the thyroid gland, although the tall cell subtype has also been documented. Follicular carcinoma is the second most common thyroid malignancy, while poorly differentiated thyroid carcinoma, anaplastic thyroid carcinoma, and medullary thyroid carcinoma are exceedingly rare [12, 15]. While most thyroid cancers associated with mature cystic teratomas occur in conjunction with struma ovarii, thyroid carcinomas can also develop in mature cystic teratomas without fulfilling the histologic criteria for struma ovarii [16]. Our case represents one of these rare occurrences—a papillary thyroid carcinoma identified within a mature cystic teratoma of the ovary in the absence of struma ovarii.

Malignant transformation of mature cystic teratomas is often diagnosed postoperatively through pathological examination [14], as preoperative diagnosis remains challenging. These tumors may be discovered incidentally during routine gynecological examinations or imaging studies conducted for unrelated clinical concerns. When symptoms occur, they are generally attributed to the ovarian mass itself and may include abdominal or pelvic pain, abdominal distension, a palpable adnexal mass, and constipation [14], none of which are directly related to thyroid malignancy. Hyperthyroidism is a rare complication, occurring when the tumor produces excess thyroid hormone. Thyroid function tests, including serum TSH, free thyroxine (T4), and total triiodothyronine (T3), are essential for evaluating thyroid status. In cases of hyperfunctioning struma ovarii (defined as thyroid tissue constituting more than 50% of the teratoma), thyroid function tests may demonstrate suppressed TSH levels with elevated thyroid hormones. In exceptional cases, thyroid carcinoma within a mature cystic teratoma has been reported in conjunction with Graves' disease [17, 18]. Serum thyroglobulin, a glycoprotein produced exclusively by thyroid follicular cells, serve as a key tumor marker for differentiated thyroid carcinoma [19]. However, preoperative thyroglobulin measurement is generally not recommended when the thyroid gland is intact, as circulating thyroglobulin can originate from both benign thyroid tissue and carcinoma, limiting its diagnostic specificity. Following total thyroidectomy and radioactive iodine ablation, the elimination of normal thyroid tissue enhances the utility of thyroglobulin as a sensitive marker for residual, recurrent, or metastatic disease. Undetectable or low thyroglobulin levels are generally indicative of a favorable therapeutic response, while rising thyroglobulin levels may indicate disease recurrence and warrant further investigation. It is important to assess thyroglobulin antibodies concurrently, as they can interfere with thyroglobulin measurement and result in falsely low values [20]. Tumor markers such as CA125 and CA19-9 may be elevated in some cases [21]. While imaging modalities, including computed tomography and ultrasound, may suggest the presence of a mature cystic teratoma, the malignant component is often only identified upon postoperative histopathological evaluation.

The histological features characteristic of papillary thyroid carcinoma, similar to those observed in the thyroid gland, include cellular enlargement, elongation, and overlapping. The chromatin demonstrates clearing and margination. Additionally, nuclear irregularities, nuclear grooves, and nuclear pseudoinclusions are commonly seen. Psammoma bodies are frequently identified within the tumor [22]. Immunohistochemical analysis is a valuable diagnostic tool, with positive staining for markers such as thyroglobulin, thyroid transcription factor-1 (TTF-1), and paired-box gene 8 (PAX8) aiding confirmation. The expression of Hector Battifora mesothelial epitope-1 (HBME-1) and cytokeratin 19 (CK19), and the absence of CD56 expression, are established immunohistochemical markers commonly found in papillary thyroid carcinoma [23]. Galectin-3, used alongside HBME-1 and CK19, aids in differentiating malignant from benign follicular-derived thyroid lesions. The BRAF V600E mutation, frequently found in papillary thyroid carcinoma, is a useful molecular marker for diagnosis, risk stratification, and potential targeted therapy [24, 25]. The proliferation marker Ki-67 is used to assess the tumor cell proliferation rate, and may indicate malignancy or aggressiveness; however, its expression can be low in certain malignant tumors [26].

When evaluating an ovarian mass exhibiting features consistent with thyroid carcinoma, the diagnostic considerations should include thyroid cancer arising within a mature cystic teratoma and ovarian metastasis from a primary thyroid carcinoma, as these conditions have distinct prognostic and therapeutic implications. Notably, ovarian metastasis of papillary thyroid carcinoma is extremely rare [27, 28]. Histologically, the absence of benign thyroid parenchyma or teratomatous components within the ovarian lesion, combined with the presence of a primary thyroid carcinoma, favors a diagnosis of metastatic papillary thyroid carcinoma rather than thyroid malignancy arising within mature cystic teratoma [29].

The management of papillary thyroid carcinoma arising in mature cystic teratomas poses a challenge due to its rarity, and the lack of a universally accepted standard of care. The initial surgical approach generally involves the removal of the ovarian tumor [3], which may vary from cystectomy to unilateral salpingo-oophorectomy, or total abdominal hysterectomy with bilateral salpingo-oophorectomy. The extent of surgery is determined by factors, including the patient's age, desire for future fertility, and the presence of extra-ovarian spread, such as capsular invasion, adhesions, ovarian surface involvement, peritoneal fluid, or metastases. The prognosis is excellent when the tumor is confined to the ovary, exhibits no surface involvement, and remains unruptured [30]. Surgical excision may be adequate in such cases. For women desiring fertility preservation, options such as unilateral oophorectomy or cystectomy are appropriate. In this patient, who is menopausal, total hysterectomy with bilateral salpingo-oophorectomy is typically performed to minimize the risk of recurrence. Staging laparotomy may be performed following cystectomy or salpingo-oophorectomy, particularly if malignancy is unexpectedly discovered during laparoscopic surgery. This procedure allows for assessment of disease extent and guides further treatment decisions. In early-stage cases with small (<2 cm), confined tumors, papillary carcinoma histology, and no aggressive histopathological features, staging laparotomy may not be required [31]. Routine prophylactic lymph node dissection is not generally recommended, unless there is evidence of extra-ovarian spread or metastasis [30].

After initial surgery for an ovarian mass, a thyroid ultrasound is recommended to rule out concurrent primary thyroid cancer. Although the thyroid gland may appear normal, the role of thyroidectomy remains controversial, and should be considered on a case-by-case basis. Some authors recommend total thyroidectomy in most cases, particularly when high-risk features are present, such as a tumor >2 cm, extra-ovarian spread, aggressive histopathological features, or metastases [32, 33]. However, the decision should also take the patient's preferences into account, considering their individual risk profile. Total thyroidectomy provides several benefits, including the ability to perform a comprehensive pathological evaluation of the thyroid gland to rule out primary thyroid cancer with ovarian metastasis. Additionally, it allows for the use of serum thyroglobulin levels as a tumor marker for recurrence, and facilitates radioactive iodine therapy for ablation of residual thyroid tissue, thereby improving the monitoring of recurrence [34]. In cases of metastasis, radioactive iodine therapy after thyroidectomy can effectively concentrate in metastatic thyroid tissue, as thyroidectomy eliminates normal thyroid uptake, allowing the iodine to target the metastases more effectively. Furthermore, thyroidectomy facilitates TSH suppression with levothyroxine to prevent tumor growth, similar to treatment for primary thyroid cancer [31]. Conversely, some authors argue that thyroidectomy may be unnecessary in the absence of high-risk features [30]. Several authors suggest that, in the absence of aggressive features, pelvic surgery alone may be sufficient—especially considering the potential risks of thyroidectomy, including perioperative complications, general anesthesia-related issues, lifelong levothyroxine replacement, and complications such as hypoparathyroidism or recurrent laryngeal nerve injury. While thyroglobulin monitoring without thyroidectomy is less sensitive, it may still play a role in follow-up when combined with clinical assessment and imaging [35]. Management of these complex cases requires a multidisciplinary team, including gynecologists, pathologists, endocrinologists, and oncologists, to ensure comprehensive care.

The prognosis of thyroid carcinoma arising in mature cystic teratoma is favorable, with 5-year survival at 96.7%, 10-year at 94.3%, and 20-year at 84.9% [36]. The mortality rate is low at 4.72%, with most death occurring in patients with distant metastasis [12]. Poor prognostic factors include tumors larger than 10 cm, carcinoma affecting >80% of strumal tissue, and extensive papillary carcinoma features, such as necrosis, ≥5 mitoses per 10 high-power fields, and marked cytological atypia [16]. Poorly differentiated or anaplastic thyroid carcinoma also indicates a poorer prognosis [16]. Follicular carcinoma is more likely to metastasize distantly compared to papillary thyroid carcinoma [16], while papillary carcinoma is more likely to metastasize to regional and para-aortic lymph nodes. BRAF mutations in primary thyroid cancer predict a poorer prognosis, but studies in malignant struma ovarii show they do not always correlate with poor outcomes [37, 38].

Regular surveillance using imaging, such as transvaginal ultrasound and abdominal computed tomography scans, is employed to monitor for recurrence or metastasis [39, 40]. Recurrence rates can be as high as 15%–35% [33, 41]. The average time to recurrence for papillary carcinoma is ~4–6 years [33], but it can occur as late as 21.4 years after surgery [16]. Given the rarity of this condition, the optimal duration of follow-up is not well-defined. Long-term follow-up is often recommended because recurrence is possible even many years after initial treatment, potentially exceeding 10 years [41, 42] and possibly up to 20 years [33].

## 4. Conclusions

We presented a rare case of papillary thyroid carcinoma identified within a mature cystic teratoma of the ovary in a 62-year-old woman. Currently, there is a lack of consensus on postoperative management. After a careful evaluation to determine the aggressive behavior of the ovarian tumor and to exclude metastasis, the option of not undergoing thyroidectomy and radioactive iodine ablation may be considered.

## Data Availability

The data that support the findings of this study are available from the corresponding author upon reasonable request.
